# Smiles-based bioactivity prediction through molecular encoder selection and data augmentation

**DOI:** 10.1186/s13321-026-01251-0

**Published:** 2026-07-06

**Authors:** Ju Hyung Lee, Seongik Choi, Utku Ozbulak, Joris Vankerschaver, Wesley De Neve

**Affiliations:** 1 Center for Biosystems and Biotech Data Science, Ghent University Global Campus, Incheon, South Korea; 2 Department of Applied Mathematics, Computer Science and Statistics, Ghent University, Ghent, Belgium; 3 Computational Data Sciences Department, George Mason University Korea, Incheon, South Korea; 4 Department of Electronics and Information Systems, Ghent University, Ghent, Belgium

**Keywords:** ASK1, ChemBERTa, IC_50_ Regression, SMILES

## Abstract

**Abstract:**

Quantitative prediction of inhibitor potency can accelerate early-stage drug discovery. Recently, data-driven approaches have gained widespread interest in drug discovery, as evidenced by a growing number of benchmarking challenges and open competitions. In this context, we developed a machine learning-based methodology that can find the most effective way of predicting IC_50_ values against ASK1 from SMILES, for *“Jump AI(.py) 2025: 3rd AI Drug Discovery Competition”*, hosted by the Korea Pharmaceutical and Bio-Pharma Manufacturers Association (KPBMA) on the Dacon platform. Applying our methodology achieved the highest overall predictive performance among all participating teams. Beyond this competition setting, we present a compact SMILES-based modeling workflow comprising (i) a pre-trained encoder, (ii) regression models, (iii) data augmentation, and (iv) hyperparameter tuning. We systematically compared molecular representations from sequence- and graph-based models, including ChemBERTa-2 and MolCLR. Across encoder–regressor combinations, ChemBERTa-77 M-MLM embeddings paired with support vector regression (SVR) yielded the strongest predictive performance. Embedding-level mix-up augmentation and SVR hyperparameter tuning further improved predictive performance. Our findings highlight that careful SMILES preprocessing and encoder selection have a critical influence on IC_50_ values and provide a reproducible benchmark for single-target bioactivity prediction, thus contributing to a more efficient drug discovery process.

**Scientific Contribution** In this study, we propose a machine learning methodology for predicting the IC_50_ values of ASK1 inhibitors from SMILES representations, with a systematic comparison of molecular encoders and regression models. Our results show that the use of suitable encoder-regressor pairs together with embedding-level mix-up augmentation improves model generalizability without requiring SMILES-level augmentation. This strategy would be particularly useful for settings with imbalanced labels or limited data, and could be applied more broadly to IC_50_ prediction for other kinase inhibitors.

**Graphical Abstract:**

**Figure:**
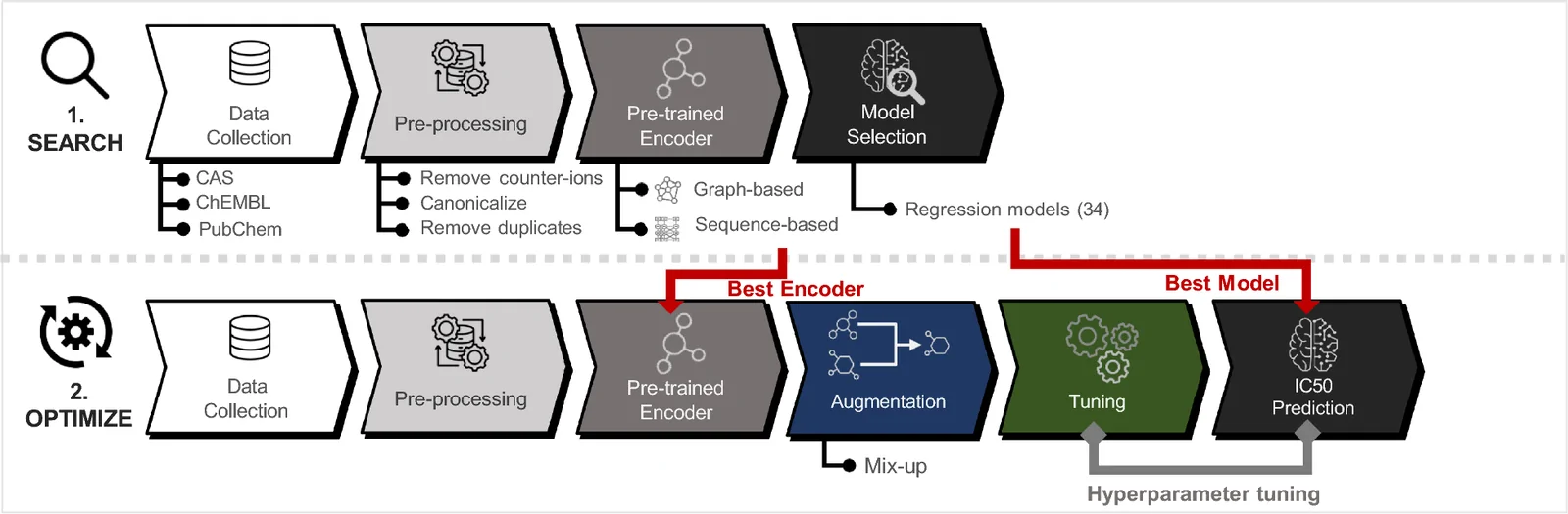

## Introduction

Apoptosis signal-regulating kinase 1 (ASK1), also known as mitogen-activated protein kinase kinase kinase 5 (MAP3K5), is a serine/threonine kinase that mediates stress-induced apoptosis and inflammation through the p38 mitogen-activated protein kinase (MAPK) and c-Jun N-terminal kinase (JNK) pathways [1]. Dysregulation of ASK1 signaling has been implicated in neurodegenerative, cardiovascular, and metabolic disorders, making ASK1 a promising therapeutic target [2, 3].

ASK1 acts as an upstream MAP3K that is activated by oxidative and endoplasmic reticulum stress and propagates these cues to the p38 MAPK and JNK cascades. Its activity is tightly regulated by redox-sensitive protein interactions and phosphorylation events, enabling rapid transitions between inactive and active states in response to cellular stress [4]. Owing to its conserved catalytic domain, ASK1 is amenable to small-molecule inhibition, most commonly via ATP-competitive binding, making potency metrics such as the half-maximal inhibitory concentration (IC_50_) directly relevant for lead optimization [5]. Nevertheless, optimizing ASK1 inhibitors remains challenging because subtle structural modifications can substantially alter hinge-binding patterns and other key interactions, motivating data-driven approaches to capture complex structure-potency relationships [6].

Accurate estimation of the IC_50_ values of small-molecule ASK1 inhibitors can substantially accelerate early-stage drug discovery by reducing experimental screening costs [7]. Traditional quantitative structure-activity relationship (QSAR) modeling relies on handcrafted molecular descriptors that may fail to capture complex and contextual interactions among substructures [8, 9]. Recent AI-based models, such as ChemBERTa, represent Simplified Molecular Input Line Entry System (SMILES) strings as dense contextual embeddings, allowing direct learning from chemical sequences [10, 11].

In this study, we present a concise yet complete evaluation of IC_50_ prediction for ASK1 inhibitors using SMILES-based molecular representations. Specifically, we investigate the effects of (i) a pre-trained encoder, (ii) regression models, (iii) data augmentation, and (iv) hyperparameter tuning. Our objective is to identify a minimal yet reproducible pipeline for prediction of inhibitor potency that balances methodological simplicity and predictive accuracy. The solution we present in this work is the winning solution for the Jump AI(.py) 2025 competition.

### Jump AI(.py) 2025: 3rd AI drug discovery competition

The solution presented in this study corresponds to the winning approach of the Jump AI(.py) 2025 competition. This competition was hosted by the Korea Pharmaceutical and Bio-Pharma Manufacturers Association (KPBMA) and ran from July 7 to August 25, during which each participating team was allowed up to three submissions per day via the Dacon platform [12]. In total, 502 teams participated, with the shared objective of advancing AI-driven methodologies for early-stage drug discovery.

## Methods

### Dataset and evaluation

The training set with ASK1 inhibitor data was constructed by integrating data from multiple sources, including CAS [13], ChEMBL [14], and PubChem [15].

A total of 28,071 SMILES sequences were obtained from multiple sources, including 3,452 sequences from the CAS database, 824 sequences from the ChEMBL database, and 23,795 sequences from PubChem.

In addition to the training set, the competition provided a separate test set containing 127 SMILES sequences, supplied by an external company (Yuhan Corporation). The labels for this test set were not disclosed. Of these, 40% were allocated to a public test subset for real-time evaluation on the public leaderboard (LD), while the remaining 60% constituted a private test subset used to determine the final ranking at the end of the competition. Importantly, the private test results were revealed only for the final submitted model; therefore, private LD scores were available for only a single model configuration.

The final predictive performance on the test set was determined using a competition-specific metric. First, A denotes the RMSE normalized by the dynamic range of the true pIC_50_ values, and B denotes the squared Pearson correlation ($R^2$) between the true and predicted pIC_50_ values:

1 $$\begin{aligned} A= & \frac{\textrm{RMSE}(y, \hat{y})}{\max (y) - \min (\hat{y})}, \end{aligned}$$

2 $$\begin{aligned} B= & \textrm{Pearson}(y,\hat{y})^{2}, \end{aligned}$$

where *y* and $\hat{y}$ denote vectors of true and predicted pIC_50_ values, respectively. The final score was then computed as follows:

3 $$\begin{aligned} \text {Score} = 0.4 \times (1 - \min (A, 1)) + 0.6 \times B, \end{aligned}$$

Lower values of A and higher values of B contribute to a higher overall score.

### Data preprocessing

**Fig. 1:**
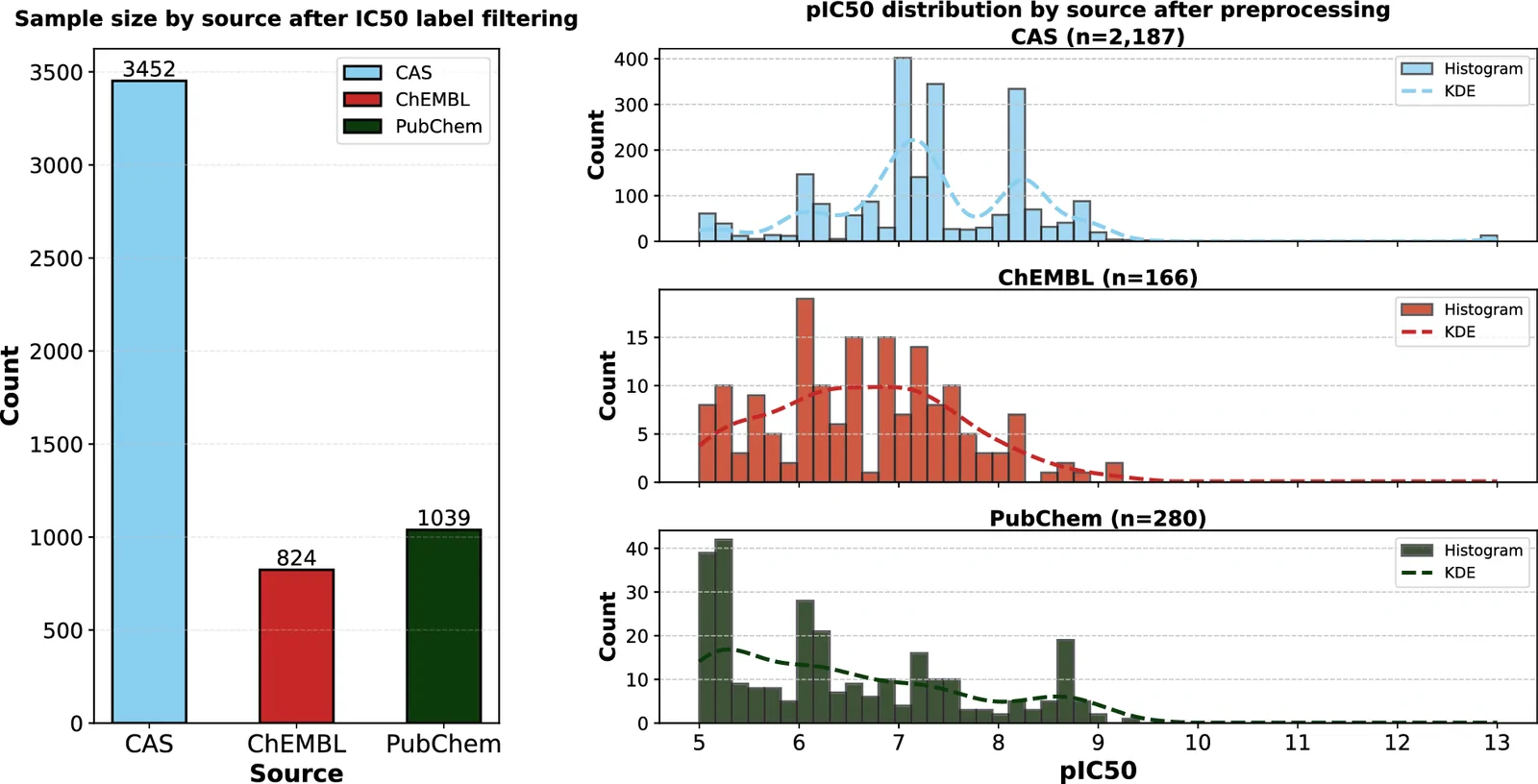
Sample size by source after IC_50_ label filtering (left) and pIC_50_ distribution by source after preprocessing (right)

As described in Fig. 1, all datasets except PubChem contain experimentally measured IC_50_ values. Accordingly, only PubChem samples with available IC_50_ annotations were retained prior to preprocessing. SMILES sequences were standardized via canonicalization, during which counterions and salt fragments were removed using RDKit [16]. Subsequently, all IC_50_ values were converted to nanomolar units and transformed into pIC_50_ values ($-\log _{10}[M]$) to ensure consistency across data sources. After the transformation, samples with pIC_50_ values below 5, which were considered inactive, were filtered out [17]. For canonical SMILES entries associated with multiple reported pIC_50_ values, the data were aggregated by selecting the minimum value. The mean was not used because averaging repeated experimental measurements would yield an artificial value rather than a directly observed assay result, and thus it would potentially introduce additional noise into the dataset. The maximum was also excluded because it increased the number of highly potent samples (e.g., pIC_50_ >9), whereas extremely high potency values corresponding to sub-nanomolar activity may be more susceptible to experimental uncertainty or reporting variability. Accordingly, the minimum value was adopted as a conservative aggregation strategy to reduce the influence of potentially overestimated potency measurements.

The raw data from PubChem contained several label types. In addition to IC50, it also included EC50, Ki, Kd, Km, and even unlabeled entries. To ensure label consistency across data sources, only samples with IC50 values were selected, resulting in 1,039 entries out of 23,795. After preprocessing, 2,704 unique and standardized SMILES strings remained, corresponding to approximately 9.6% of the entries in the original dataset. A large portion of irrelevant data in PubChem (shown in Fig. S1) and removal of duplicate entries contributed to such an abrupt reduction of data. Subsequently, after filtering the data based on an acceptable pIC_50_ threshold (≥5.0) within the range of active compounds, a final set of 2,633 SMILES strings was obtained.

### Pre-trained encoder

SMILES representations must be converted into numerical features to serve as inputs for machine learning models. Different feature extraction methods encode molecular information in distinct ways, and their suitability may vary depending on the prediction task. In this context, molecular foundation models, which are deep learning models primarily designed for learning informative representations, are widely applied as feature extractors to chemical compounds.

Based on the considerations above, we evaluated multiple feature extraction strategies, including the generation of Morgan fingerprints and the derivation of feature vectors from molecular foundation models [18], namely MolCLR (graph-based) and ChemBERTa-2 (sequence-based).

ECFP4s were computed as 2048-dimensional binary vectors that encode the presence of specific substructures or chemical features [19]. This representation captures topological patterns such as functional groups, ring systems and local connectivity, and serves as a baseline encoder.

MolCLR, a graph-based molecular foundation model, was employed to extract graph-level representations learned through self-supervised pretraining. MolCLR performs contrastive learning on molecular graphs using augmentation strategies such as atom masking and bond deletion [20]. Two backbone architectures were employed:

- Graph Convolutional Network (GCN): aggregates neighborhood information through normalized averaging.
- Graph Isomorphism Network (GIN): performs injective sum aggregation followed by an MLP, enabling higher expressive power for distinguishing subtle structural variations in molecular graphs [21].

Both GCN- and GIN-based variants of the pretrained MolCLR model produce 512-dimensional continuous feature vectors that capture a compact representation of the molecular graph.

Transformer-based sequence representations were obtained from ChemBERTa-2, a SMILES-based molecular foundation model. Unlike graph-based encoders, ChemBERTa-2 operates directly on the SMILES syntax and captures long-range dependencies through its transformer architecture. Two pretrained variants were employed:

- Masked Language Modeling (MLM): learns structure-aware sequence representations by predicting masked SMILES tokens in a self-supervised manner.
- Multi-Task Regression (MTR): incorporates supervised property prediction to embed task-relevant chemical features into the learned representations [22–24].

Both variants of ChemBERTa-2 generate 384-dimensional continuous embeddings that encode a compact, sequence-level representation of molecular structures. For clarity, we refer to the MLM-pretrained and MTR-pretrained models as ChemBERTa-77 M-MLM and ChemBERTa-77 M-MTR, respectively.

### Model selection

Due to the limited number of submissions allowed in the competition, it was not feasible to evaluate all combinations of the five encoders and 34 regression models through leaderboard submissions. To address this limitation, we first performed encoder selection using a Random Forest (RF) regressor as the baseline model. By keeping the regressor fixed and varying only the encoder, we compared the quality of the extracted molecular representations. Using the representations obtained from the selected encoder, a wide range of machine learning-based regression algorithms was then applied to predict pIC_50_ values. A 5-fold cross-validation (CV) strategy was adopted to ensure robustness and generalizability [25]. Predictive performance was assessed using root mean square error (RMSE) and the coefficient of determination ($R^2$). The best-performing regressor was subsequently selected based on the average predictive performance across all five folds. In addition, the encoder was kept frozen and was not fine-tuned to reduce the risk of overfitting given the limited amount of available training data.

## Results

### Feature extraction

As summarized in Table 1, irrespective of the aggregation strategy or embedding type, ECFP4 (Morgan fingerprint with radius = 2) achieved the highest average predictive performance in 5-fold cross-validation with a fixed RF regressor. Specifically, ECFP4 representations exhibited the highest mean $R^2$ (0.72), the lowest mean RMSE (0.57), and the highest mean LD score (0.81).

Although ECFP4 representations demonstrated superior predictive performance on the training set across all aggregation strategies, their predictive performance on the external test set was noticeably weaker, indicating limited generalization ability. In contrast, ChemBERTa-77 M-MLM embeddings, the second best performer, achieved stronger predictive performance on the external test set, demonstrating that their feature representations are more robust and capable of capturing chemically meaningful patterns beyond the training distribution.

**Table 1:** Predictive performance of different feature extractors, using a fixed RF regressor with 5-fold cross-validation

Encoder	Training set			Test set
	mean RMSE ↓	mean $R^2\,\uparrow$	Mean LD score ↑	LD score ↑
ECFP4	**0**.**57** ± **0**.**04**	**0.72 ** ± ** 0.02**	**0.81 ** ± ** 0.01**	0.34
GCN	0.78 ± 0.04	0.49 ± 0.03	0.66 ± 0.02	0.31
GIN	0.75 ± 0.03	0.52 ± 0.04	0.68 ± 0.03	0.38
MLM	0.65 ± 0.05	0.64 ± 0.02	0.76 ± 0.02	**0**.**51**
MTR	0.69 ± 0.04	0.59 ± 0.03	0.72 ± 0.02	0.34

Test set scores were obtained through submission to the competition server. Training set results are reported as mean ± standard deviation. The best results for each metric are highlighted in bold

The gap in predictive performance between training and test sets led us to prioritize generalization over training performance. Although ECFP4 representations exhibited the strongest overall predictive performance across all conditions in the training set, the corresponding models showed substantially weaker generalization on the external test set. Due to this gap in predictive performance, we ultimately selected the ChemBERTa-77 M-MLM encoder, which demonstrated more stable and reliable predictive performance between training and test sets.

**Fig. 2:**
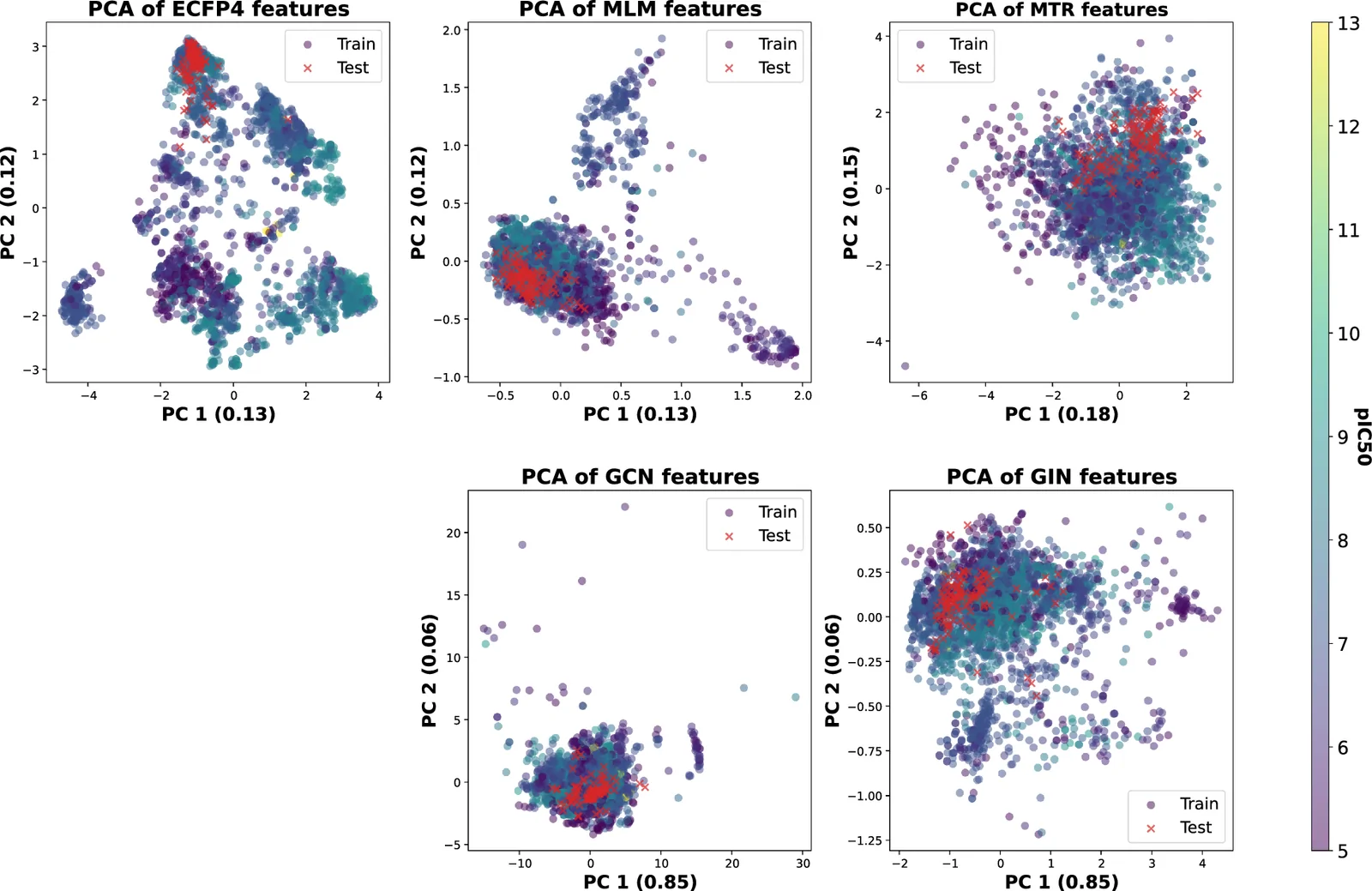
Principal component analysis (PCA) of molecular embeddings extracted from the ChemBERTa-2 (MLM and MTR) and MolCLR (GCN and GIN) models. Points are colored by pIC_50_ values, with training and test samples indicated separately

Moreover, we compared the distribution of the data points (training and test compounds) in the embedding space of each encoder using PCA projections (Fig. 2). The test set largely overlaps with the training set in the two-dimensional PCA projection, suggesting that the test compounds are not entirely separated from the training compounds in the embedding space. While MolCLR-based embeddings (GCN and GIN) exhibit a higher variance contribution in PC1 (and PC1+PC2) than ChemBERTa-2 embeddings, this geometric property did not correspond to improved predictive performance (Table 1).

### Machine learning model comparison

After selecting ChemBERTa-77 M-MLM as the encoder, the entire dataset was used as the training set to comprehensively evaluate various regressors. We evaluated 34 different regressors, and Table 2 presents the top-10 performing models based on cross-validation. LightGBM (LGBM) achieved the best results within the training set with an RMSE of 0.64±0.05 and $R^2$ of 0.65±0.02, followed by ExtraTrees (RMSE=0.65±0.05, $R^2$=0.64±0.03) and Support Vector Regression (SVR, RMSE= 0.67±0.05, $R^2$=0.62±0.03).

**Table 2:** Predictive performance of the top-10 regression models from a total of 34 models

Models	Mean RMSE ↓	Mean $R^2\,\uparrow$	Public LD score ↑
LGBM	**0**.**64** ± **0**.**05**	**0**.**65** ± **0**.**02**	0.56
ExtraTrees	0.65 ± 0.05	0.64 ± 0.03	—
SVR	$\underline{0.67 \pm 0.05}$	$\underline{0.62 \pm 0.03}$	**0.62**
NuSVR	0.67 ± 0.05	0.62 ± 0.03	0.60
RandomForest	0.67 ± 0.05	0.62 ± 0.03	0.51
XGBoost	0.67 ± 0.04	0.62 ± 0.01	0.46
GradientBoosting	0.69 ± 0.05	0.60 ± 0.02	—
Bagging	0.70 ± 0.04	0.58 ± 0.01	—
BayesianRidge	0.73 ± 0.03	0.55 ± 0.02	—
LassoLarsIC	0.73 ± 0.03	0.54 ± 0.03	—
⋮			

The ChemBERTa-77 M-MLM encoder was used, and 5-fold cross-validation was applied. Results are reported as mean ± standard deviation. Public LD Scores were obtained through submission to the competition server; a dash(—) indicates that the corresponding score was not available from the competition. The best-performing results for each metric are highlighted in bold, while the final selected model is additionally indicated by underlining

Due to the limited number of submission attempts, not all combinations could be evaluated on the test set, resulting in several missing public LD scores in Table 2. While LGBM and ExtraTrees, two tree-based ensemble methods, occupied the top rankings in cross-validation, SVR and NuSVR demonstrated peak generalization performance on the external test set, achieving public LD scores of 0.62 and 0.60, respectively. The discrepancy between cross-validation and test performance highlights the importance of evaluating model performance beyond training metrics, and suggests that regularization properties of SVR may provide better robustness to small data sizes, and to distribution shifts between training and test data.

### Data augmentation and hyperparameter tuning

Given the highly imbalanced distribution of pIC_50_ values across a wide numerical range, data augmentation became essential to improve model robustness. Although SMILES enumeration resulted in a slight performance improvement, its effectiveness was limited: the degree of enumeration is inherently random, difficult to control, and incapable of generating samples in unrepresented pIC_50_ regions [26]. For example, if no data exist within the 9–11 pIC_50_ range, SMILES enumeration cannot compensate for this gap because it does not alter the underlying label distribution.

**Fig. 3:**
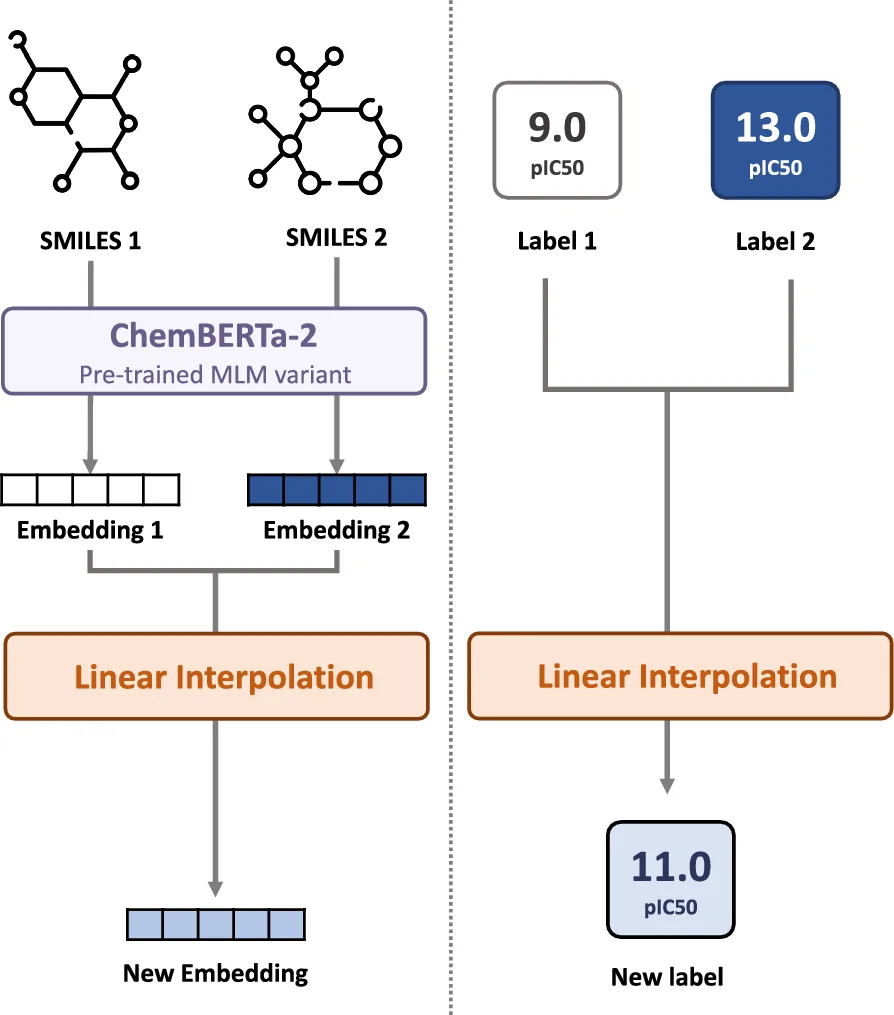
Illustration of our application of mix-up augmentation between embedding vectors. Two samples are linearly interpolated (averaged) to generate a synthetic sample, improving coverage of underrepresented label regions in the dataset

**Fig. 4:**
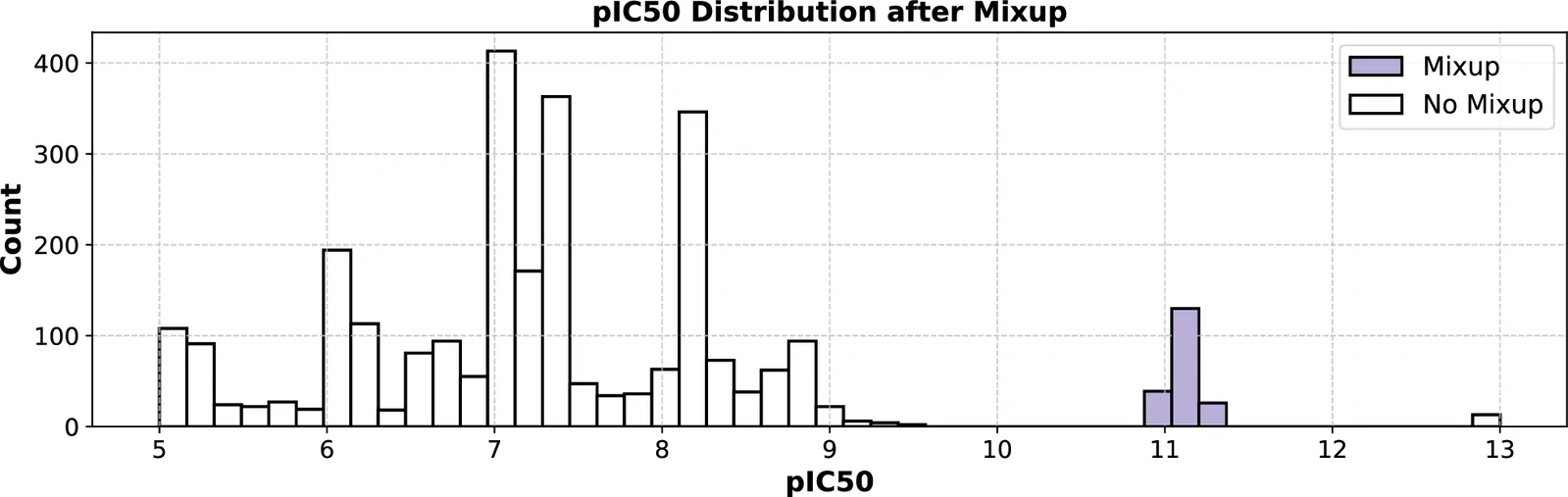
pIC_50_ distribution before and after mix-up data augmentation. Samples with pIC_50_ of approximately 11 were added after augmentation

To address this limitation, we applied a mix-up augmentation strategy directly to the embedding vectors, targeting underrepresented pIC_50_ intervals [27]. Let ${\boldsymbol{x}} \in \mathbb {R}^{384}$ denote a molecular embedding and $y \in \mathbb {R}$ the associated pIC_50_ value. Given two training pairs $(x_1,y_1)$ and $(x_2,y_2)$, synthetic pairs $(\tilde{x},\tilde{y})$ are constructed as

4 $$\begin{aligned} \tilde{{\boldsymbol{x}}}= & \lambda {\boldsymbol{x_1 }}+ (1 - \lambda ){\boldsymbol{x_2}} \end{aligned}$$

5 $$\begin{aligned} \tilde{y}= & \lambda y_1 + (1 - \lambda )y_2 \end{aligned}$$

with mixing coefficient $\lambda \in [0,1]$. In this study, we fixed λ to 0.5. Figure 3 illustrates this construction. In particular, samples with pIC_50_ 9-10 were paired with samples with pIC_50_ 13, and all possible pairs were used to generate synthetic samples with pIC_50_ around 11. Rather than merely increasing sample size, this approach enriched the label space and yielded more substantial gains in predictive performance compared to naive data duplication or SMILES enumeration. As a result, 195 samples with pIC_50_ range between 11.02 and 11.26 were generated (Table 3, Fig. 4) Under the augmented data distribution, the SVR model further enhanced predictive performance as illustrated in Table 4.

**Table 3:** Summary of synthetic samples generated via mix-up augmentation

New samples	Mean pIC_50_	pIC_50_ range
195	11.12	11.02-−11.26

**Table 4:** Impact of mix-up augmentation on predictive performance. The RMSE values are reported as mean ± standard deviation. The best results for each metric are highlighted in bold

	RMSE ↓	Public LD score ↑
No mix-up	0.67 ± 0.05	0.61
Mix-up	**0**.**64** ± **0**.**03**	**0**.**72**

**Table 5:** Effect of SVR hyperparameter tuning under the mix-up augmented setting

Setting	Hyperparameters	RMSE ↓	Public LD score ↑
Default	$C=1.0,\; \epsilon =0.1$	**0**.**64** ± **0**.**03**	0.72
Tune 1	$C=0.9,\; \epsilon =0.1$	0.65 ± 0.02	**0**.**74**
Tune 2	$C=0.8,\; \epsilon =0.1$	0.65 ± 0.02	**0**.**74**
Tune 3	$C=0.8,\; \epsilon =0.005$	0.66 ± 0.02	**0**.**74**
Tune 4	$C=0.5,\; \epsilon =0.005$	0.68 ± 0.03	0.73

The RMSE values are reported as mean ± standard deviation across five folds. The best results for each metric are highlighted in bold

Compared to the default configuration (kernel = rbf, *C* = 1.0, ϵ = 0.1, gamma = scale), reducing both the regularization parameter *C* and the ϵ-insensitive loss led to consistent improvements in predictive performance. The gains in predictive performance obtained through hyperparameter optimization are summarized in Table 5. Model selection through hyperparameter tuning was performed based on 5-fold cross-validation, and the predicted values for the test set generated by the selected models were submitted. As a result, the highest public leaderboard score, based on the official competition leaderboard, was achieved with *C* = 0.8 and ϵ = 0.005.

**Fig. 5:**
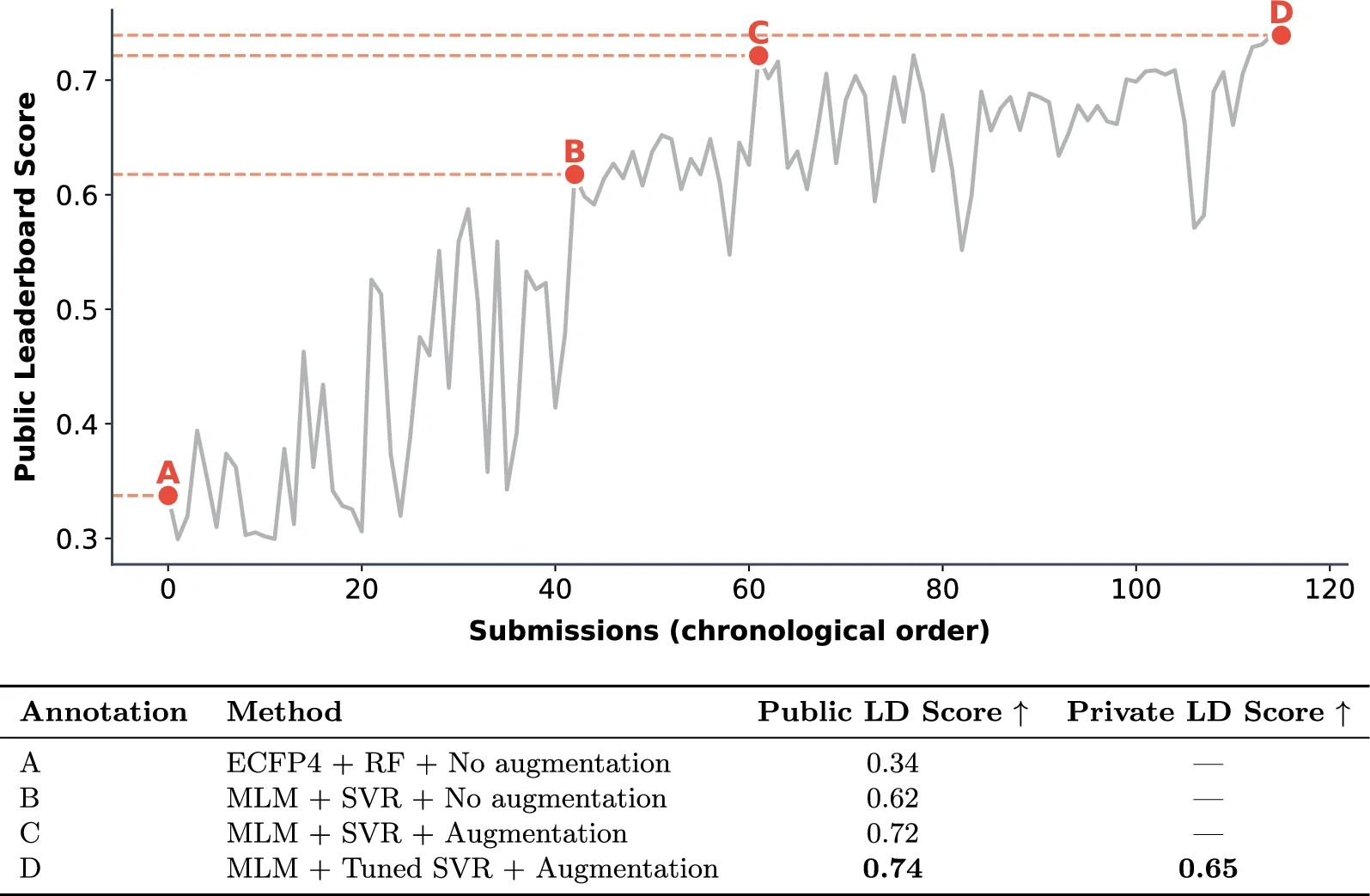
Predictive performance progression and corresponding methods. Among the evaluated configurations, annotation D was selected as the final submitted model; therefore, it was the only configuration assessed on the private leaderboard. A dash (—) indicates that the corresponding private leaderboard score was not available from the competition. The best results for each metric are highlighted in bold

To illustrate the model development process during the competition, Fig. 5 summarizes the chronological progression of our public leaderboard scores across all submitted experiments. Each point on the curve represents one submission from our team, ordered by submission time. The annotated points (A) to (D) indicate representative milestone submissions corresponding to key modeling decisions: (A) baseline, (B) encoder and regressor pair selection, (C) data augmentation, and (D) hyperparameter tuning. Based on these experimental comparisons, we found that combining mix-up augmentation, which was designed to populate underrepresented regions of both chemical space and the pIC_50_ label distribution, with SVR hyperparameter tuning consistently improved predictive performance. Among all evaluated settings, the best-performing model used ChemBERTa-77 M-MLM as the encoder with SVR, together with mix-up data augmentation and hyperparameter tuning. This model achieved the highest public LD score (0.74) and resulted in a private LD score of 0.65 (Fig. 5).

### Ablation study: Tox24 challenge

Given the restricted accessibility of the dataset used in this study, we conducted an ablation study on an additional publicly available dataset and corresponding task to improve reproducibility and verify that the observed trends in predictive performance are not dataset-specific.

The Tox24 Challenge is an international computational toxicology competition designed to evaluate the predictive performance of modern machine learning approaches in predicting the in vitro activity of chemicals  [28]. Specifically, building upon the earlier Tox21 initiative [29], the Tox24 Challenge focuses on predicting the binding activity of chemical compounds to transthyretin (TTR) [30], a transport protein associated with thyroid hormone regulation. The released benchmark comprises 1,512 compounds with TTR binding measurements, split into training (1,012), leaderboard (LD, 200), and blind (300) sets. The chemical structures are represented as SMILES, primarily derived from the ToxCast libraries, and encompass heterogeneous entities such as mixtures, salts, and metal-containing compounds. During the challenge, the leaderboard subset supported preliminary evaluation; in a later phase, organizers released leaderboard labels and allowed participants to merge those compounds (with labels) into the training pool for refinement, whereas the blind subset remained undisclosed until the challenge ended and defined the official RMSE-based ranking. In our ablation study, the released leaderboard labels were merged into the training pool following this protocol. Therefore, all configurations were trained on the combined 1,212 compounds (training and leaderboard sets), and the blind set served as the sole held-out evaluation set.

**Table 6:** Tox24 benchmark ablation for configurations (A) to (D), reporting 5-fold CV RMSE on the training set, RMSE on the leaderboard set, and RMSE on the blind set

Annotation	Method	CV RMSE ↓	LD set RMSE ↓	Blind set RMSE ↓
A	ECFP4 + RF + No augmentation	26.9 ± 0.3	9.8	25.0
B	MLM + LGBM + No augmentation	26.8 ± 1.3	**2**.**7**	24.9
C	MLM + LGBM + Augmentation	25.7 ± 0.5	**2**.**7**	23.1
D	MLM + Tuned LGBM + Augmentation	**25.7 ± 0.3**	3.8	**22**.**7**

Lower RMSE indicates better agreement with experimental readouts. Bold values indicate the best result per column

Table 6 reports the Tox24 ablation following the same (A) to (D) progression as in the main study. This experiment was not designed as a fully optimized Tox24 submission. Rather, we applied the workflow with minimal challenge-specific modification to assess whether the staged improvements observed in the ASK1 study also appear on an independently accessible benchmark. Because Tox24 differs from ASK1 in target biology, assay characteristics, data composition, and challenge design, the absolute predictive performance level and the optimal regressor are not expected to transfer directly. Consistently, whereas SVR paired best with MLM embeddings in the ASK1 study, LightGBM performed best among the regressors evaluated under the same protocol for Tox24.

Under this constrained verification setting, blind-set RMSE improved progressively from configuration (A) to (D). Adding mix-up augmentation in (C) produced the largest blind-set gain in this ladder, and tuning the regressor in (D) further reduced the blind RMSE. The 5-fold CV RMSE followed the same downward trend, decreasing from 26.9 in (A) to 25.7 in (D), providing a held-out estimate consistent with the blind-set progression. Therefore, the Tox24 experiment should be interpreted as a transparency and reproducibility check rather than as a claim of Tox24-specific optimization or state-of-the-art predictive performance. Despite this limitation, the consistent improvement across the ablation sequence supports the main methodological claim that the proposed combination of molecular representation learning, augmentation, and regressor tuning can improve external-set performance beyond the restricted ASK1 dataset.

## Discussion

This study highlights how SMILES preprocessing, feature extraction, model selection, and data augmentation collectively influence model performance in the prediction of IC_50_ values for ASK1 inhibitors. Among all tested molecular representations, the combination of ChemBERTa-77 M-MLM embeddings with SVR achieved the highest predictive performance on the external test set. Because predictive performance varied substantially across both molecular encoders and regressors, the choice of encoder-regressor pair appears to play a decisive role in determining predictive accuracy.

In Tables 1 and 5, discrepancies were observed between the cross-validation and test-set results. This discrepancy should be interpreted cautiously because the test set was not generated by splitting the full dataset, but was instead provided as an external set from *Yuhan Corporation* based on in-house experiments. In terms of representation geometry, the PCA-based chemical-space analysis (Fig. 2) indicates that the test compounds are largely embedded within the training-set manifold, suggesting that the test set is not entirely outside the training chemical space. However, this apparent overlap should be interpreted cautiously, because PCA provides a low-dimensional global projection and does not necessarily reflect local compound-level structural similarity between individual training and test compounds. To further examine this point, we calculated pairwise Tanimoto similarities between the test and training compounds as described in Table S3. The average similarity across the full test-training similarity matrix was 0.23, indicating that most test-training pairs were structurally dissimilar. In addition, the average nearest-neighbor similarity for each test compound was 0.53, with the highest nearest-neighbor similarity reaching 0.88. These results suggest that, although some test compounds had close analogues in the training set, the overall local structural similarity between the two sets was limited. Therefore, the PCA overlap may reflect global embedding-level similarity rather than strong compound-level similarity across the two splits.

Because the individual test-set labels were not disclosed, we could not directly assess whether label shift occurred. Therefore, the discrepancy between cross-validation and test-set performance should not be attributed to covariate shift alone. A plausible hypothesis is that the test compounds occupy a partially overlapping chemical space while exhibiting different pIC_50_ distributions due to differences in assay conditions, experimental protocols, or measurement contexts. Accordingly, we interpret the gap in predictive performance as a possible consequence of both limited local structural similarity and potential label shift, rather than as definitive evidence of covariate shift alone.

Furthermore, although ChemBERTa-77 M-MTR has been shown to outperform ChemBERTa-77 M-MLM across multiple downstream fine-tuning tasks [22], the pretrained MLM embeddings yielded superior predictive performance in our IC_50_ prediction setting. This suggests that ASK1 inhibitor potency prediction is less dependent on the task-specific molecular descriptors incorporated into the MTR training objectives and may instead benefit more from the structural and contextual patterns captured by MLM-based representations.

Notably, MolCLR-based embeddings (GCN/GIN) exhibited a larger fraction of explained variance in the first principal components (PC1 and PC1+PC2), implying a more compressed low-dimensional structure. However, this variance concentration did not translate into improved predictive performance (Table 1), underscoring that PCA compactness is not necessarily aligned with task-relevant information for potency prediction. This is plausible because PCA is unsupervised and emphasizes directions of maximum variance, which may be driven by variance-dominant factors, such as molecular size or frequent structural motifs, rather than potency-relevant signals. Consequently, task-informative features may reside in lower-variance directions that are not captured by PC1/PC2.

In this study, we selected these molecular representation models to reflect three widely adopted paradigms [31]: a fingerprint-based approach (ECFP4), a SMILES-based transformer approach (ChemBERTa-2), and a graph-based approach (MolCLR). This selection was intended to cover conceptually distinct and commonly used representation strategies. We acknowledge that other molecular representation models, such as MolFormer [32] and MolEncoder [33], are also relevant and may offer complementary strengths. However, due to the constraints of the competitive setting and the substantial improvement of ChemBERTa-2 MLM over baseline ECFP4, we prioritized the initially selected representation models instead of conducting additional exhaustive comparison. Future work will focus on evaluating a broader range of molecular representation models to better assess their predictive performance and generalizability in SMILES-based IC_50_ prediction tasks.

For the PubChem dataset, most data points were filtered out during the preprocessing steps. In particular, the “Unlabeled” category contains SMILES strings without associated experimental values, which limited its direct use in supervised learning. Nonetheless, these unlabeled molecules may still have considerable value. Instead of merely using them to recover missing labels and enlarge the supervised dataset, future research could explore their relationship to the synthetic samples generated through mix-up augmentation. Because those augmented samples were obtained by interpolating embedding vectors from a large pretrained model, the unlabeled molecules may help reveal whether the generated samples occupy chemically meaningful regions of the latent space.

In addition to the choice of pretrained embeddings, the mix-up-based data augmentation strategy contributed notably to improvements in predictive performance [27]. Although previous studies have shown that SMILES enumeration can expand the explored chemical space [26], it cannot populate empty regions between existing samples. Moreover, applying mix-up directly at the SMILES string level can generate chemically invalid molecules due to violations of SMILES grammar. To address these limitations, we applied mix-up in the embedding space, which avoids syntactic issues and enables smooth interpolation within the learned latent chemical manifold. This embedding-level augmentation produced more robust representations and improved generalization on the external test set.

This improved generalization is particularly relevant in regions where the original data distribution is sparse. In our dataset, no compounds were observed in the pIC_50_ range between 10 and 13, resulting in an apparent gap in the activity distribution. Such a pattern may suggest the presence of an activity cliff, where small structural differences lead to large changes in biological activity [34]. In this context, mix-up augmentation in the embedding space can partially alleviate this issue by introducing intermediate representations (e.g., around pIC_50_ ∼11), thereby improving the continuity of the pIC_50_ distribution. However, these interpolated samples are synthetic and may not correspond to real molecular structures, and should therefore be interpreted with caution.

Apart from mix-up augmentation, hyperparameter optimization of the SVR model further mitigated biases arising from imbalance in the original dataset, particularly the scarcity of samples in the pIC_50_ range of 10-12 [35]. This, in turn, improved the ability of the model to generalize across the augmented label space.

Because the proprietary CAS-derived fraction of the training data and the held-out competition test labels are not publicly redistributable, independent replication using the full Jump AI(.py) 2025 dataset is constrained. To provide verifiable evidence for the proposed workflow despite this limitation, we repeated the (A) to (D) ablation on the Tox24 challenge (Table 6), which uses a publicly available dataset. The blind-set labels were released after the challenge closed, and the official ranking was based on blind RMSE. Predictive performance improved from configuration (A) to configuration (D), although the best-performing regressor differed from that used in the ASK1 study.

On Tox24, the absolute predictive performance remains modest relative to the challenge front-runners. The final configuration (D) achieved a blind RMSE of 22.7 (Table 6), whereas the published leaderboard reported top blind RMSE values near 20.5 - 20.8 [36]. In terms of cross-validation performance (Table 6), our 5-fold CV RMSE decreased from configuration (A) to configuration (D), consistent with the blind-set improvement. For reference, the nine individual teams analyzed by Cirino et al. [37] reported train-set CV RMSEs ranging from 20.8 to 26.5; our configuration (D) at 25.7 falls within this range, near the lower tier. Although our blind RMSE of 22.74 does not match the strongest reported results, published accounts of the challenge indicate that the top-performing entries typically relied on consensus or ensemble predictors combining multiple models, rather than on a single regressor. In contrast, our goal was not to construct a Tox24-specific optimized submission, but to retain a single explicit pipeline for ablation and external validation. Closing the remaining gap in predictive would likely require analogous ensemble, consensus, or multi-representation strategies, which we consider beyond the scope of this study.

## Conclusions

In summary, careful data preprocessing and appropriate selection of representation are critical for accurate IC_50_ prediction, a key component of early-stage drug discovery. This work provides a concise yet comprehensive methodology for SMILES-based potency prediction that integrates data preprocessing, molecular representation, data augmentation and regression modeling. Future work will explore additional molecular representation strategies and explore mix-up augmentation more thoroughly to further extend the proposed methodology.

## Supplementary Information

Supplementary file 1.

## Data Availability

The code used in this study, together with the SMILES entries obtained from ChEMBL and PubChem, is available at https://github.com/SeongIk-Choi/2025_Dacon. The competition details are available at https://dacon.io/competitions/official/236530. However, the CAS-derived fraction of the training data and the held-out external test set are not publicly redistributable due to the competition data policy and copyright restrictions. Therefore, the central results based on the full Jump AI(.py) 2025 dataset cannot be fully reproduced from the shared materials alone. To support reproducibility, we additionally provide the materials for the public Tox24 ablation study in the same GitHub repository.

## Abbreviations

AI: Artificial Intelligence
ASK1: Apoptosis signal-regulating kinase 1
ATP: Adenosine Triphosphate
CAS: Chemical abstracts service
CV: Cross-validation
ECFP4: Extended-Connectivity Fingerprint 4
GCN: Graph Convolutional Network
GIN: Graph Isomorphism Network
IC50: Half-maximal inhibitory concentration
JNK: C-Jun N-terminal kinase
KPBMA: Korea Pharmaceutical and Bio-Pharma Manufacturers Association
LD: Leaderboard
MAPK: Mitogen-activated protein kinase
MAP3K5: Mitogen-activated protein kinase kinase kinase 5
MLM: Masked language modeling
MLP: Multi-Layer Perceptron
MolCLR: Molecular Contrastive Learning of Representations
MTR: Multi-Task Regression
PCA: Principal component analysis
pIC50: Negative base-10 logarithm of IC_50_
QSAR: Quantitative structure-activity relationship
R2: Coefficient of determination
RF: Random Forest
RMSE: Root Mean Square Error
SVR: Support Vector Regression
NuSVR: Nu-Support Vector Regression
XGBoost: EXtreme Gradient Boosting
