# Supplementary material for: Smiles-based bioactivity prediction through molecular encoder selection and data augmentation
Source: J Cheminform. 2026 Jul 6;18:137. doi: 10.1186/s13321-026-01251-0 (PMC13625499; doi:10.1186/s13321-026-01251-0)
Supplement: Supplementary file 1 [file 13321_2026_1251_MOESM1_ESM.pdf]

Supplementary Material:
SMILES-Based Bioactivity Prediction through Molecular Encoder Selection and Data Augmentation

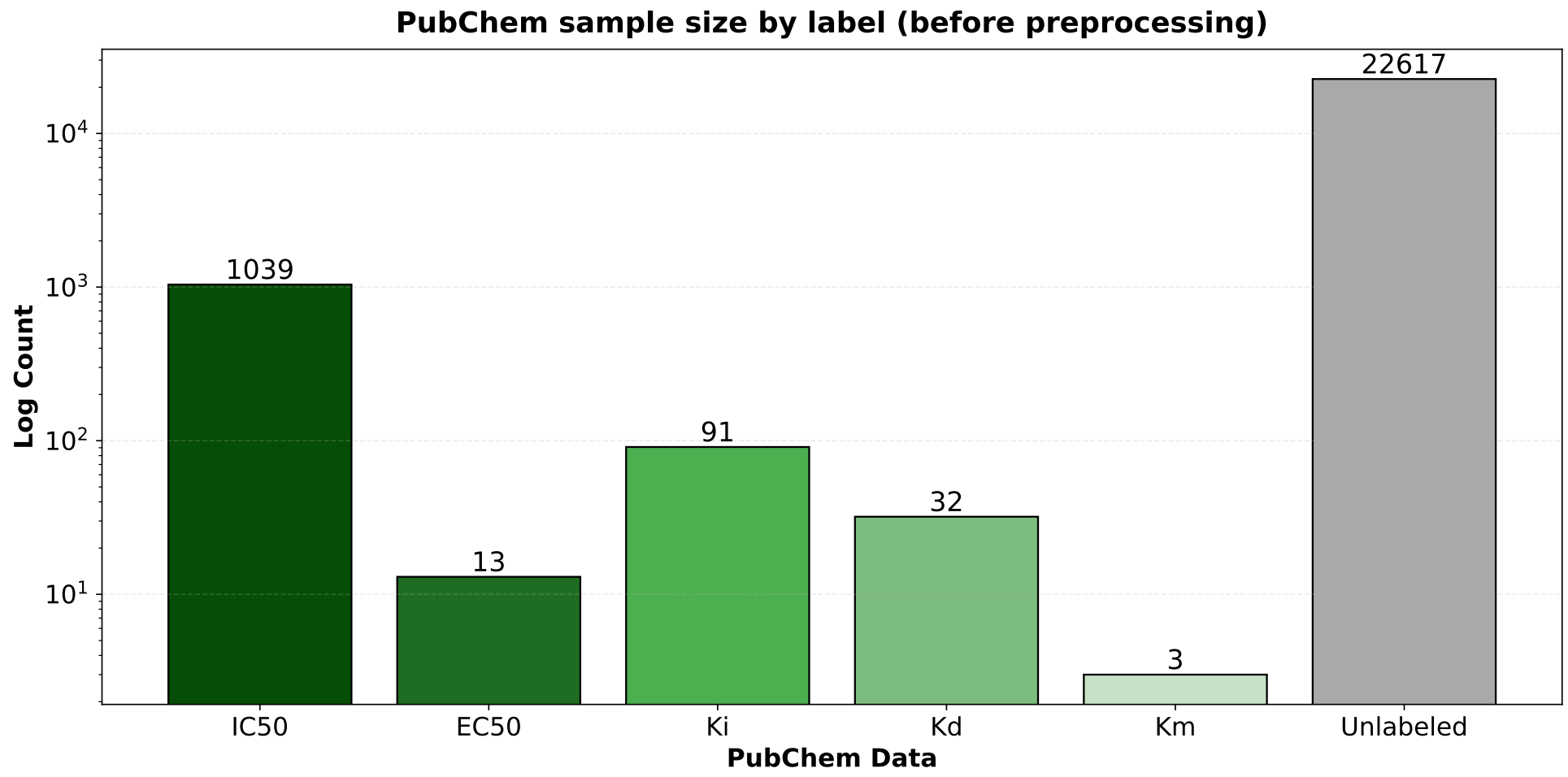

Fig. S1: PubChem label distribution before preprocessing, with y-axis in log scale. Large portion of "Unlabeled" data in PubChem contributed to huge reduction of data after preprocessing.

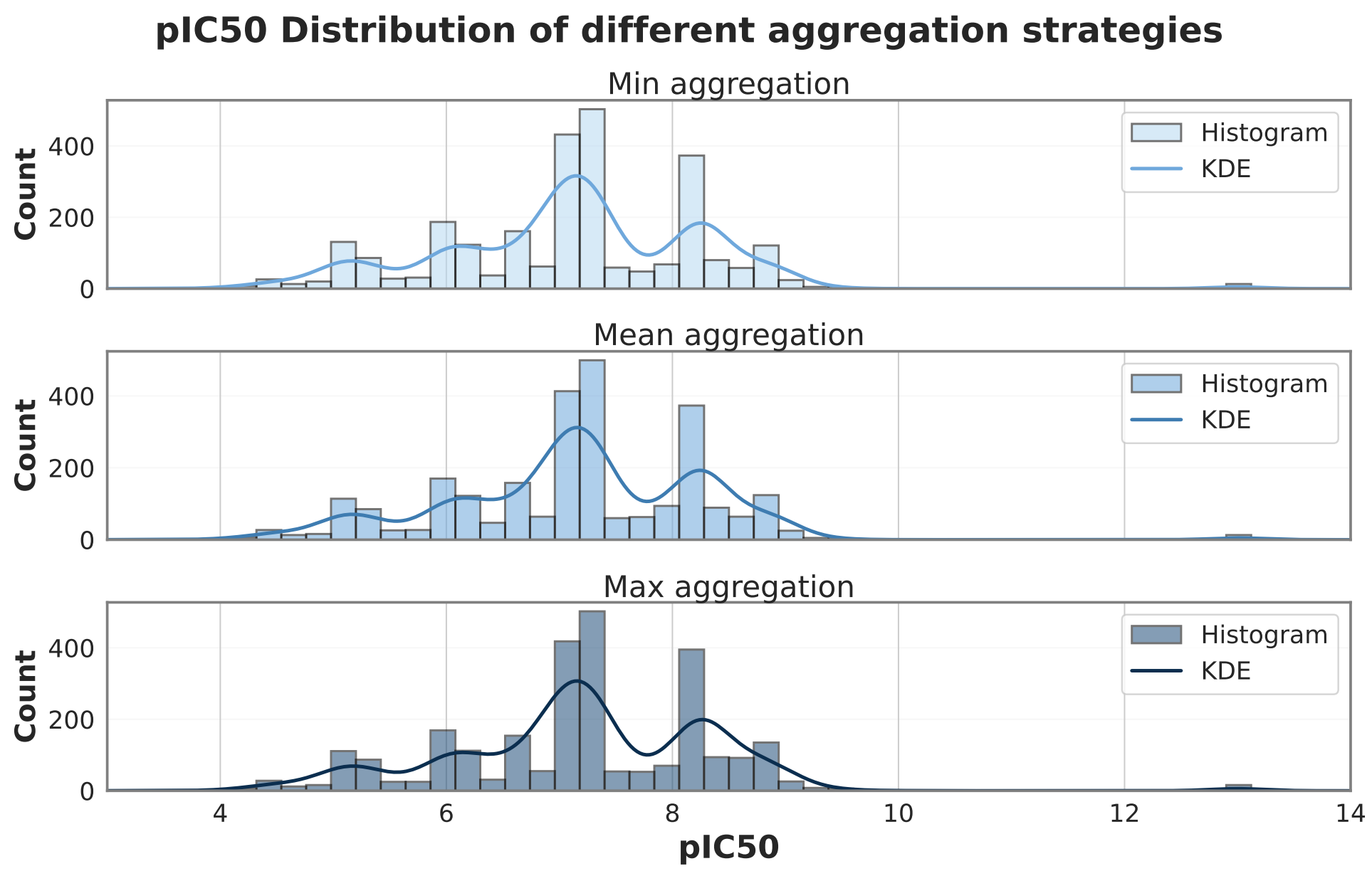

Fig. S2: Comparison of data distribution under different duplicate aggregation strategies: minimum (top), mean (center) and maximum (bottom).

Table S1: Explained variance ratio (%) of the first two principal components for each encoder.

**Encoder**	**PC1 (%)**	**PC2 (%)**	**PC1+PC2 (%)**
ECFP4	13.0	12.0	25.0
MLM	13.0	12.0	25.0
MTR	18.0	15.0	33.0
GCN	85.0	6.0	91.0
GIN	85.0	6.0	91.0

Table S2: Full result of model performance comparison of 34 regression models (5-fold CV).

Model	RMSE ↓	R^2 ↑
LGBMRegressor	0.64 ± 0.05	0.65 ± 0.02
ExtraTreesRegressor	0.65 ± 0.05	0.64 ± 0.03
SVR	0.67 ± 0.05	0.62 ± 0.03
NuSVR	0.67 ± 0.05	0.62 ± 0.03
KNeighborsRegressor	0.67 ± 0.04	0.62 ± 0.01
RandomForestRegressor	0.67 ± 0.05	0.62 ± 0.03
XGBRegressor	0.67 ± 0.04	0.62 ± 0.01
GradientBoostingRegressor	0.69 ± 0.05	0.60 ± 0.02
BaggingRegressor	0.70 ± 0.04	0.58 ± 0.01
BayesianRidge	0.73 ± 0.03	0.55 ± 0.02
LassoLarsIC	0.73 ± 0.03	0.54 ± 0.03
PoissonRegressor	0.74 ± 0.04	0.54 ± 0.02
Ridge	0.75 ± 0.03	0.52 ± 0.03
HuberRegressor	0.75 ± 0.04	0.52 ± 0.03
TransformedTargetRegressor	0.76 ± 0.03	0.51 ± 0.03
LinearRegression	0.76 ± 0.03	0.51 ± 0.03
MLPRegressor	0.76 ± 0.04	0.50 ± 0.05
GammaRegressor	0.78 ± 0.05	0.48 ± 0.02
LinearSVR	0.78 ± 0.04	0.48 ± 0.04
TweedieRegressor	0.78 ± 0.05	0.48 ± 0.02
OrthogonalMatchingPursuit	0.79 ± 0.04	0.47 ± 0.03
AdaBoostRegressor	0.83 ± 0.04	0.42 ± 0.02
SGDRegressor	0.86 ± 0.15	0.37 ± 0.16
DecisionTreeRegressor	0.87 ± 0.06	0.35 ± 0.05
ExtraTreeRegressor	0.93 ± 0.05	0.26 ± 0.11
PassiveAggressiveRegressor	0.96 ± 0.07	0.20 ± 0.15
ElasticNet	1.09 ± 0.05	-0.01 ± 0.09
Lasso	1.09 ± 0.05	-0.01 ± 0.09
DummyRegressor	1.09 ± 0.05	-0.01 ± 0.09
LassoLars	1.09 ± 0.05	-0.01 ± 0.09
QuantileRegressor	1.09 ± 0.05	-0.01 ± 0.09
RANSACRegressor	3.16 ± 0.30	-7.69 ± 2.15
GaussianProcessRegressor	6.79 ± 0.07	-38.41 ± 3.75
KernelRidge	7.21 ± 0.05	-43.38 ± 3.73

Table S3: Summary of pairwise Tanimoto similarities between the test compounds and training compounds. The full matrix contains similarities between 127 test compounds and 2,633 training compounds. For each test compound, the nearest-neighbor similarity was defined as the maximum Tanimoto similarity to any training compound.

**Metric**	**Value**
Number of test compounds	127
Number of training compounds	2,633
Mean similarity across the full matrix	0.24
Minimum similarity across the full matrix	0.02
Maximum similarity across the full matrix	0.88
Mean nearest-neighbor similarity	0.53
Median nearest-neighbor similarity	0.52
Minimum nearest-neighbor similarity	0.45
Maximum nearest-neighbor similarity	0.88

Table S4: Distribution of nearest-neighbor Tanimoto similarities for the test compounds. For each test compound, the maximum Tanimoto similarity to all training compounds was used.

**Nearest-neighbor Tanimoto range**	**Number of test compounds**	**Percentage**
0.4–0.5	35	27.6 %
0.5–0.6	80	63.0 %
0.6–0.7	9	7.1 %
0.7–0.8	2	1.6 %
0.8–0.9	1	0.8 %
